# Evaluation of hybrid models based on image segmentation and inference for pig weight estimation

**DOI:** 10.3389/frai.2026.1876396

**Published:** 2026-09-14

**Authors:** Miguel Angel Valles-Coral, Kelvin Lleins Rojas-Córdova, Lloy Pinedo, Richard Injante, Pierre Vidaurre-Rojas, Jorge Saavedra-Ramírez, Fernando Ruiz-Saavedra, Williams Ramirez

**Affiliations:** 1 Grupo de Investigación en Inteligencia Artificial, Facultad de Ingeniería de Sistemas e Informática, Universidad Nacional de San Martín, Tarapoto, Peru; 2 Grupo de Investigación Transformación Digital Empresarial, Facultad de Negocios, Universidad Privada Norbert Wiener, Lima, Peru; 3 Grupo de Investigación Gestión ATEC, Facultad de Ciencias Económicas, Universidad Nacional de San Martín, Tarapoto, Peru; 4 Grupo de Investigación Ganadería Amazónica Sustentable, Escuela Profesional de Ingeniería en Zootecnia, Facultad de Ingeniería y Ciencias, Universidad Nacional Autónoma de Alto Amazonas, Yurimaguas, Peru; 5 Grupo de investigación, Innovación Tecnológica en Ingeniería Civil y Arquitectura-INTEICA, Escuela de Ingeniería Civil, Facultad de Ingeniería Civil y Arquitectura, Universidad Nacional de San Martin, Tarapoto, Peru; 6 Grupo de Investigación en Manejo Forestal Sostenible, Escuela Profesional de Agronomía, Facultad de Ciencias Agrarias, Universidad Nacional de San Martín, Tarapoto, Peru

**Keywords:** feature extraction, instance segmentation, precision livestock farming, scale normalization, supervised regression

## Abstract

**Introduction:**

Accurate weight estimation in pig production is essential for optimizing management, feeding, and commercialization decisions; however, traditional weighing methods are invasive, time-consuming, and prone to operational errors. This study proposes a non-invasive computer vision–based approach to estimate pig weight under real farm conditions in San Martín, Peru.

**Methods:**

A dataset of 3,800 lateral images paired with their corresponding ground-truth weights was collected. A computational pipeline was implemented, including geometric standardization, instance segmentation using YOLOv8n-seg, and feature extraction through EfficientNet-B0. The resulting embeddings were used as input for supervised regression models (SVR, XGBoost, and CatBoost), evaluated using repeated stratified cross-validation and an independent test set, with MAE, RMSE, and R² as performance metrics. Statistical comparisons were conducted using the Friedman test followed by Wilcoxon post hoc analysis with Holm correction.

**Results:**

The results demonstrated strong predictive performance, with the SVR model achieving the best results (RMSE = 2.68 kg, MAE = 1.81 kg, R^2^ = 0.85), showing statistically significant differences compared to the other models.

**Discussion:**

These findings indicate that combining computer vision techniques with models capable of capturing non-linear relationships effectively models the relationship between animal morphology and body weight, providing a low-cost, non-invasive solution applicable to real-world production systems and supporting the advancement of precision livestock farming.

## 1 Introduction

Pig production is a fundamental activity within the agricultural sector, and its efficiency largely depends on the accurate monitoring of animal weight, a key parameter for evaluating growth, welfare, feeding strategies, and commercial decision-making (Pierozan et al., 2020; Blavi et al., 2021). However, manual weighing remains the predominant method in farms despite its limitations: it requires direct handling, induces stress in animals, increases the risk of injuries, and may introduce human errors that affect the reliability of weight records (Ogawa et al., 2024; Ramirez et al., 2022). Moreover, recent studies have shown that handling-induced stress negatively impacts both animal health and meat quality (Kikuti et al., 2023; Brando and Norman, 2023).

In livestock production systems, factors related to management, feeding, and production practices significantly influence the physical and productive characteristics of animals, as well as the quality of derived products. Experimental studies conducted under real field conditions have demonstrated that these variables can generate substantial variability in production outcomes, highlighting the need for proper process control across the agricultural value chain (Cañar-Ramos et al., 2024).

The demand for automated and non-invasive measurement systems becomes even more evident in regions experiencing rapid growth in the pig production sector, such as Peru, where production has steadily increased in recent years (MIDAGRI, 2024; Redacción 3tres3 Latinoamérica, 2024). In the San Martín region, pig farming represents a relevant component of the local economy (Carranza-Serna et al., 2025; INEI, 2024), creating opportunities to incorporate computer vision and machine learning technologies to enhance production processes.

Advances in artificial intelligence have enabled the development of methods capable of estimating animal weight using 2D images, 3D data, or RGB-D inputs, reducing human intervention while improving accuracy (Ma et al., 2024; Navarro-Cabrera et al., 2025; Nguyen et al., 2023). Modern approaches typically combine automatic body segmentation, morphometric feature extraction, and supervised regression models. For instance, methods based on Mask R-CNN, YOLOv8, ConvNeXtV2, or hybrid models have shown promising results in sheep, guinea pigs, pigs, and cattle (Canaza-Cayo et al., 2024; Ormeño-Ayala and Zapata-Ttito, 2024; Xie et al., 2024; Xu et al., 2024). In the specific case of pigs, computer vision algorithms have demonstrated the ability to segment body structures with high accuracy even in variable environments (Guvenoglu, 2023; Wang et al., 2024) and to estimate weight with average errors below 5 kg, depending on the architecture employed (Chen et al., 2023; Liu et al., 2023; Paudel et al., 2023).

Despite these advances, several technical challenges remain. Model accuracy depends on factors such as image quality, lighting variability, animal movement, partial occlusions, and the availability of sufficiently large and representative datasets (Devi Priya et al., 2022). In real farm environments, particularly when computer vision systems are deployed at larger scale or in intensive production settings, fences, barriers, surrounding animals, farm structures, and visual blind spots may partially obscure the animal body. These sources of visual interference can reduce dataset quality and negatively affect subsequent recognition, segmentation, feature extraction, and weight estimation stages. This issue has been reported in livestock systems based on deep learning, where image obstruction and dim lighting conditions can decrease recognition accuracy (Pan et al., 2023). Additionally, in small- and medium-scale farms, the cost and complexity of implementing advanced systems often limit their adoption (Busch, 2023; Molina-Benavides et al., 2025; Siegford, 2024).

In this context, integrating modern segmentation techniques such as YOLOv8 with supervised regression models, including CatBoost (Prokhorenkova et al., 2018), Support Vector Regression (Drucker et al., 1996), and XGBoost (Chen and Guestrin, 2016), represents an efficient alternative for accurately estimating pig weight in real-world environments. Segmentation enables the isolation of the animal silhouette and reduces the influence of background elements (Minaee et al., 2021; Yu et al., 2023), while supervised regression models can learn non-linear relationships between image-derived features and animal body weight (Chen et al., 2023; Jiang et al., 2024).

This study proposes a hybrid approach that combines: (1) YOLOv8n-seg-based instance segmentation, (2) feature extraction using EfficientNet-B0 (Tan and Le, 2019), and (3) supervised regression models for weight prediction. The study is conducted under real pig production conditions in Peru, aiming to contribute to the implementation of efficient, non-invasive, and scalable solutions in the region. This approach seeks to overcome the limitations of traditional methods and provide evidence on the applicability of hybrid models in open-field livestock environments, where variability in conditions can significantly affect the performance of computer vision systems.

## 2 Materials and methods

### 2.1 Data collection and dataset construction

Data collection was conducted at the “Ganadería DR” farm, located in the district of Zapatero, province of Lamas, San Martín region, Peru (6.593742° S, 76.494892° W), an area characterized by warm and humid conditions typical of the high jungle. The study was carried out between January and July 2025, encompassing planning, data acquisition and curation, hybrid model development, statistical analysis, and final evaluation. Prior to the study, formal authorization was obtained from the farm owner to access the facilities and conduct field activities.

During data acquisition, biosecurity protocols and non-invasive handling practices were applied, prioritizing animal welfare. Direct contact with the pigs was minimized, and movement toward the weighing system was guided by trained personnel, reducing stress and ensuring a controlled and safe environment, as illustrated in Figure 1.

**Figure 1:**
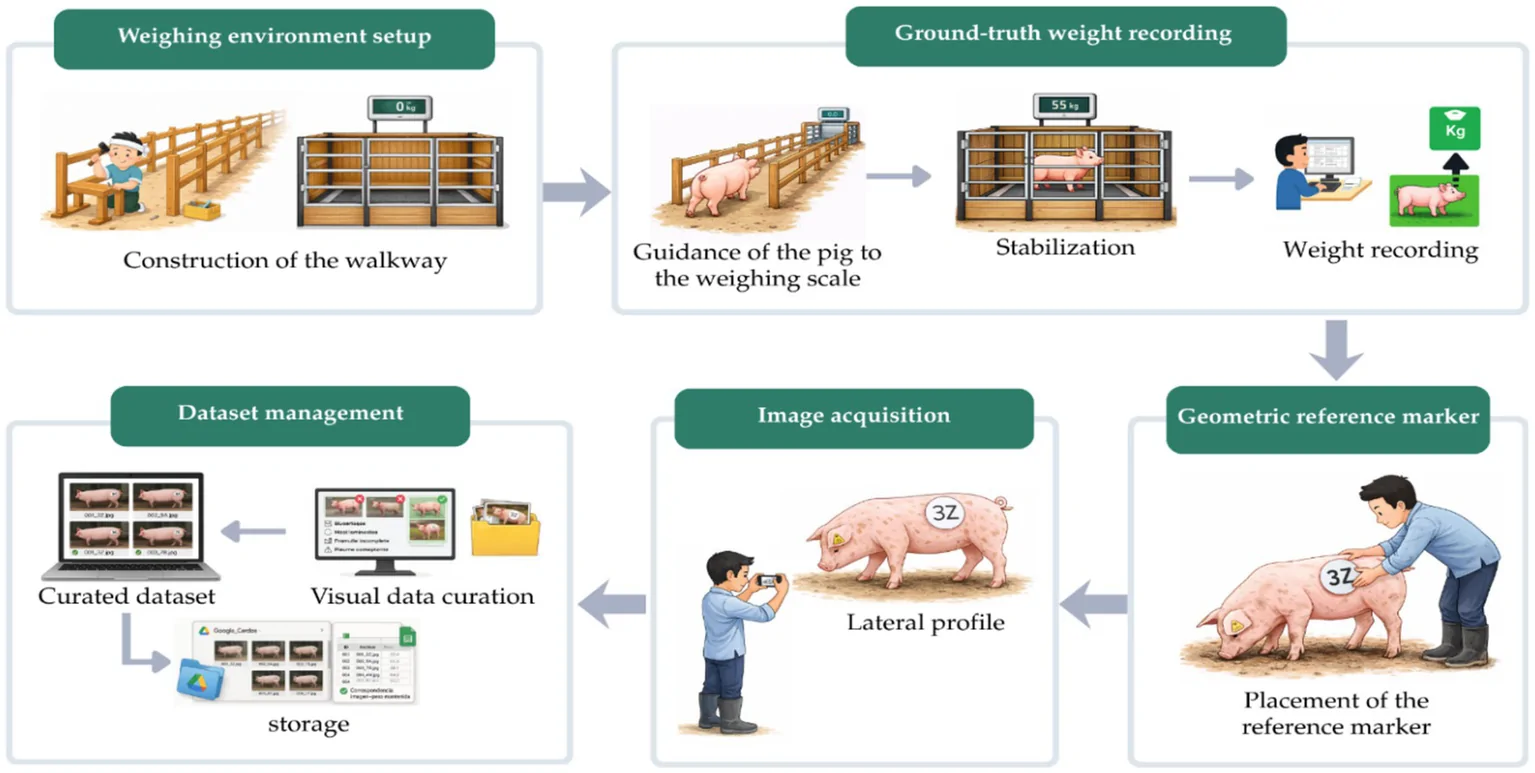
Workflow for data collection and dataset construction under farm conditions.

#### 2.1.1 Weighing environment setup

A walkway was constructed to guide the animals from the entry area to a cage-type weighing scale, enabling orderly movement and reducing external distractions. This infrastructure was designed to minimize animal stress and ensure proper positioning during weighing, in accordance with recommended handling and animal welfare practices in production systems (Ramirez et al., 2022).

The weighing system consisted of a cage-type platform (270 × 100 × 200 cm) with a reinforced metal structure and a corrugated steel surface. It was equipped with sliding doors at both ends to allow controlled animal handling. The system incorporated a high-precision electronic indicator with an LCD display and tare and hold functions, enabling reliable measurements even in the presence of animal movement, similar to automated weighing systems reported in pig production (Hou et al., 2024).

#### 2.1.2 Ground-truth weight recording

The actual body weight of each pig was recorded using the cage-type scale installed within the walkway system, which guided the animals from the entry point to the weighing area. The system calibration was verified before each data collection session. Each animal was assigned a unique identifier linked to its recorded weight (kg), ensuring traceability between the weighing process and the corresponding captured images (Hou et al., 2024).

#### 2.1.3 Geometric reference marker

After weighing and before the animal exited the scale, a circular self-adhesive vinyl marker with a diameter of 4 inches was placed on the pig’s body, as shown in Figure 2. The marker included the animal’s identifier and served a dual purpose: (i) visual identification of each specimen and (ii) a scale reference for distance and proportion normalization during preprocessing (Qin et al., 2022).

**Figure 2:**
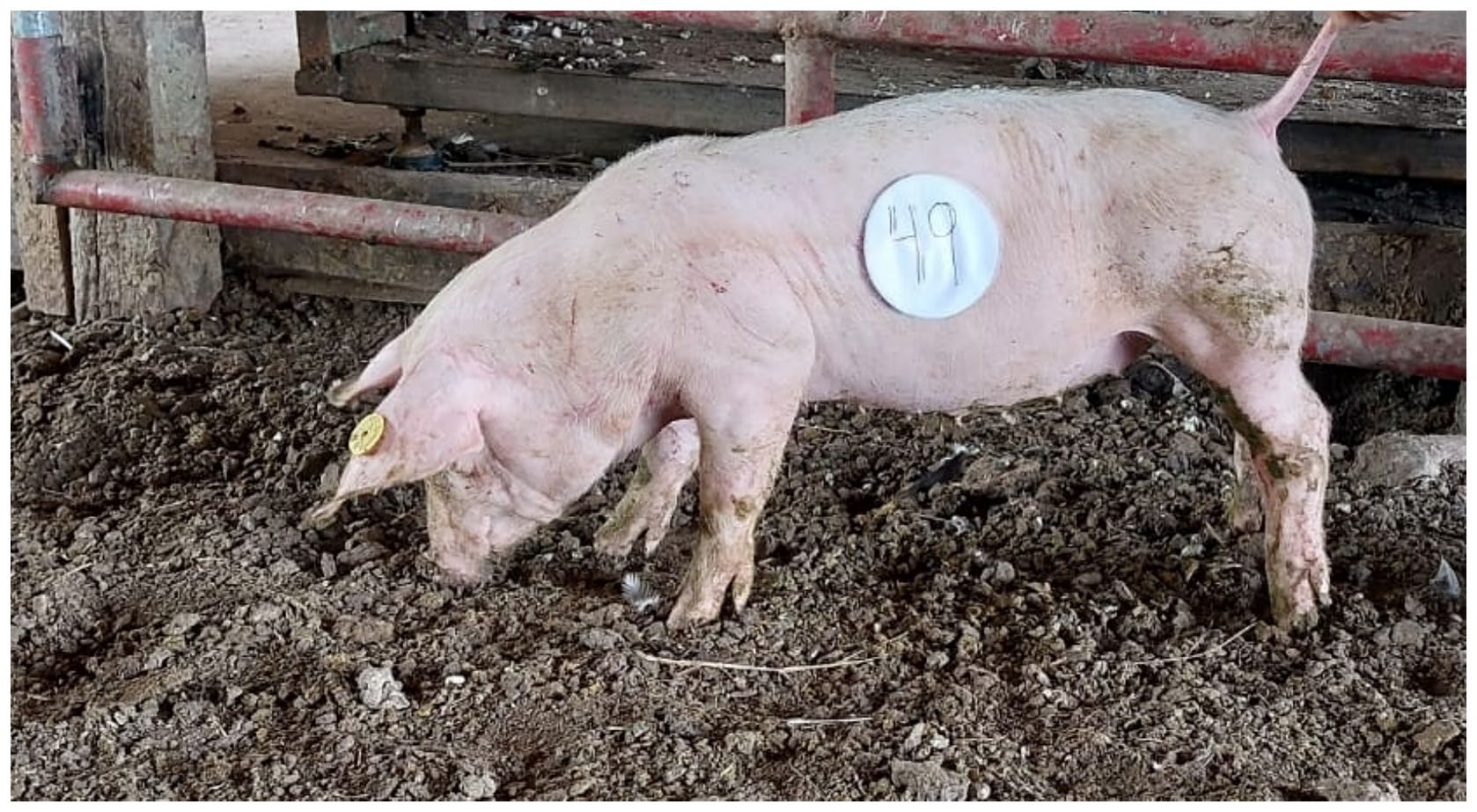
Reference marker placed on the animal.

#### 2.1.4 Image acquisition

Images were captured in RGB format using the rear main camera of a Samsung Galaxy A52s 5G smartphone, equipped with a 64 MP sensor, f/1.8 aperture, autofocus, and optical image stabilization. Image acquisition was conducted between approximately 7:00 a.m. and 12:00 p.m., recording the complete lateral profile of each pig immediately after weighing to ensure correspondence between the acquired image and the ground-truth weight. The smartphone was used in horizontal orientation, giving priority to keeping both the complete lateral body profile and the circular reference marker clearly visible within the frame, since the marker was required for subsequent geometric normalization.

The camera was positioned approximately perpendicular to the lateral plane of the animal, seeking to keep the full body within the frame while minimizing tilt, motion blur, visible occlusions, and excessive background interference that could affect marker detection or body contour delineation. A fixed capture distance was not imposed, as the study aimed to simulate real farm conditions using an accessible mobile device, in line with previous studies that have employed mobile imaging devices in livestock-related computer vision tasks (Nguyen et al., 2023; Ormeño-Ayala and Zapata-Ttito, 2024); however, variations in scale, framing, and background were subsequently addressed through preprocessing, segmentation, and image standardization. The dataset was intentionally limited to 2D lateral RGB images; therefore, dorsal images or specific backfat measurements were not included, as these correspond to a different scope related to 3D modeling or additional morphometric assessment. Background visual interference was reduced using YOLOv8n-seg-based instance segmentation, preserving the animal body region and suppressing external elements before deep feature extraction.

#### 2.1.5 Dataset management

Initially, an approximate dataset of 4,500 images was collected, each associated with its corresponding ground-truth weight. A visual curation process was subsequently applied, discarding images with blur, poor lighting, incomplete framing, incorrect image–weight pairing, severe background interference, or visible occlusions that compromised the identification of the pig body contour or the circular reference marker. The final dataset was organized in a digital repository (Google Drive), as illustrated in Figure 3, with files named using a sequential identifier while preserving correspondence with the recorded weight. The resulting dataset comprised a total of 3,800 images.

**Figure 3:**
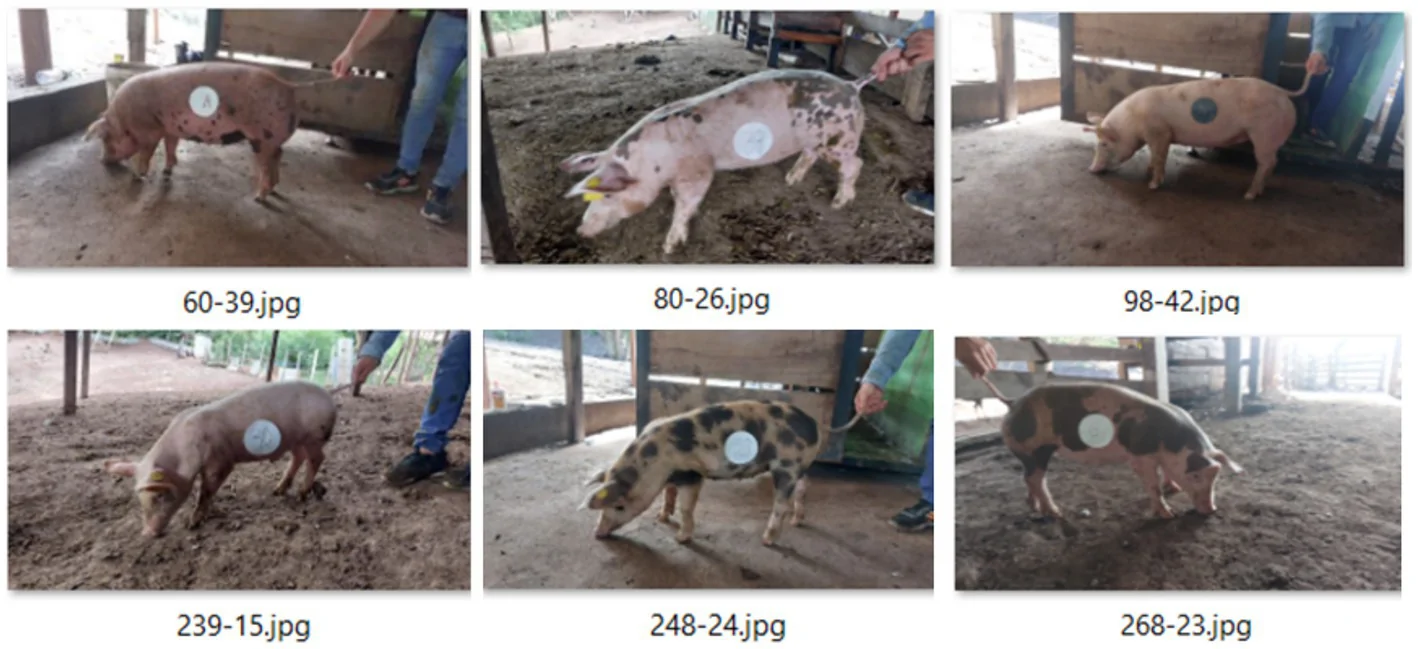
Examples of dataset images with associated identifiers and weights.

### 2.2 Computational pipeline for image processing and weight estimation

The processing of the acquired images was structured through a computational pipeline composed of multiple sequential stages aimed at estimating pig body weight. This workflow includes geometric normalization, animal body segmentation, deep feature extraction, and modeling using supervised regression techniques (Figure 4).

**Figure 4:**
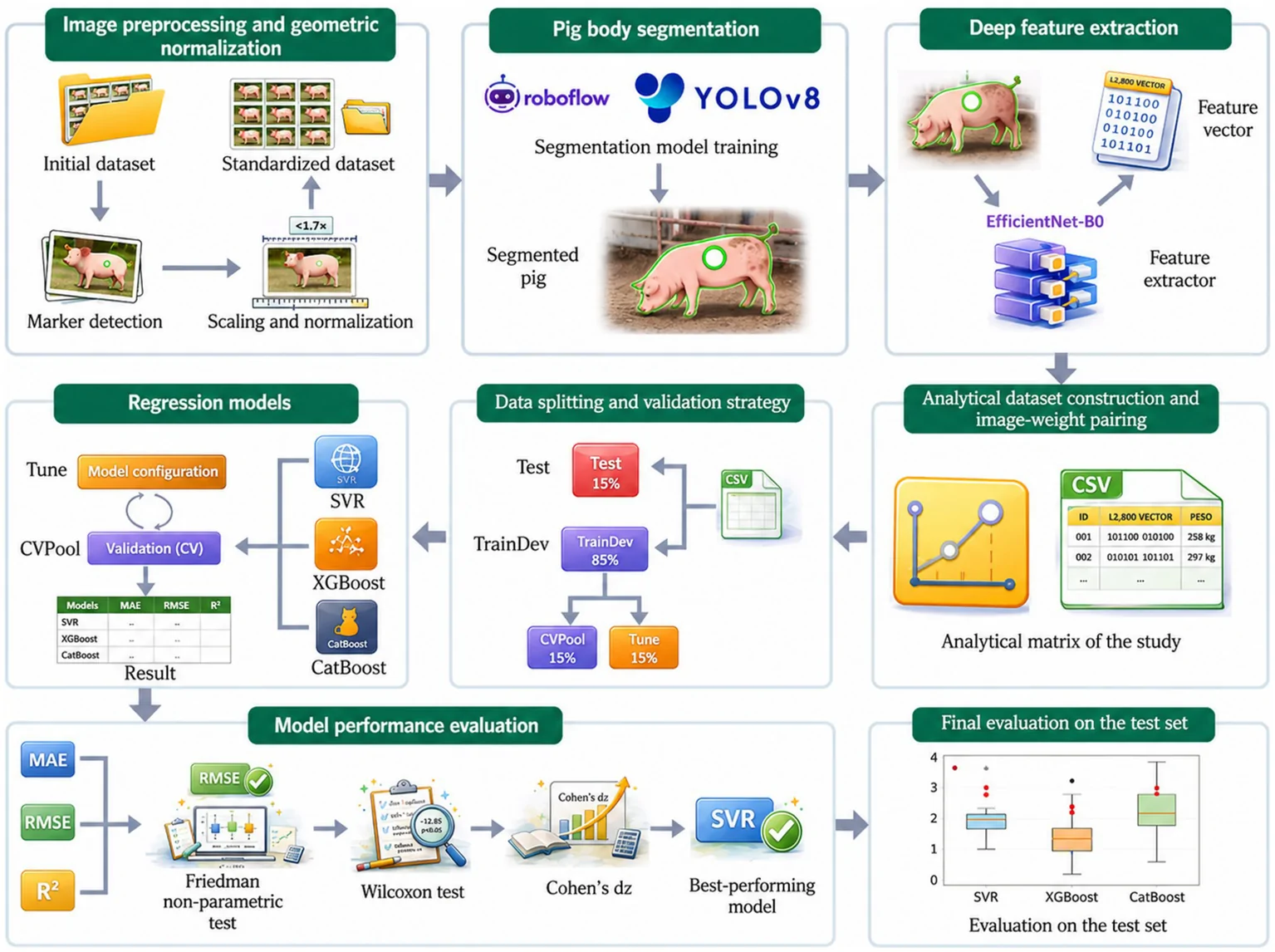
Computational pipeline for image processing and pig weight estimation.

#### 2.2.1 Image preprocessing and geometric normalization

The first stage of the pipeline consisted of geometric standardization of the images to ensure comparability across samples (Minaee et al., 2021). An orientation correction step was applied to consistently align the position of the animals within the image plane.

Subsequently, the circular reference marker placed on the animal’s body was automatically detected using a custom YOLOv8-based instance segmentation model developed specifically for this study and trained on annotated images of the marker. The predicted segmentation mask was used to determine the marker’s center and radius, with contour analysis and Hough circle detection applied when refinement was required. Based on these measurements, a scaling factor was estimated to normalize the relative dimensions of each image.

Using this factor, images were resized and adjusted to a uniform representation canvas while preserving the animal’s proportions and correcting variations caused by differences in capture distance and framing. This process reduced geometric variability across observations (Yu et al., 2023).

Finally, the preprocessed images were stored using a consistent format and naming convention, facilitating their integration with weight records and their use in subsequent stages of the pipeline.

#### 2.2.2 Pig body segmentation

Once the images were standardized, the animal body was segmented to isolate the region of interest and reduce the influence of background elements. A YOLOv8n-seg instance segmentation model was used for this stage. Since no pig-specific segmentation model adapted to the farm conditions considered in this study was available, a separate dataset of 232 pig images was manually annotated using the Roboflow platform, producing one instance-level mask of the pig body per image.

The annotated dataset was divided into 163 training images, 46 validation images, and 23 test images. The YOLOv8n-seg model was initialized from pretrained weights (yolov8n-seg.pt) and fine-tuned for 50 epochs using an input resolution of 640 × 640 pixels and a batch size of 16. The training configuration used automatic optimizer selection (optimizer = auto), with an lr0 parameter of 0.01 and a three-epoch warm-up period. The optimization objective comprised the standard YOLOv8 segmentation loss components for bounding-box regression, mask segmentation, classification, and distribution focal loss (DFL). Training and validation losses for these components were monitored throughout the 50 epochs to assess convergence and model behavior.

The training subset was used for model parameter optimization, whereas the validation subset was used to monitor model development and assess whether the resulting masks were adequate for the subsequent image-processing pipeline. The 23-image test subset remained separate from model training and validation; however, the segmentation performance reported in this study corresponds specifically to the validation subset rather than to an independent test-set evaluation. This choice reflects the role of segmentation as an intermediate preprocessing component whose purpose was to provide reliable pig-body masks for subsequent deep feature extraction, rather than as the primary predictive endpoint of the study.

After model development and validation, the exported segmentation checkpoint was applied to the 3,800 images of the study dataset to generate the pig-body masks used in the subsequent processing stages. The predicted masks delineated the visible pig silhouette, while pixels outside the mask were treated as non-informative background and suppressed before deep feature extraction, thereby reducing visual interference from the surrounding environment (Minaee et al., 2021).

#### 2.2.3 Deep feature extraction

From the segmented images, feature representations were extracted using a pretrained convolutional neural network. Previous research has shown that CNN-based representations can capture visual information related to pig body structure and use it for body weight estimation (Liu et al., 2023). EfficientNet-B0 was selected as the feature extractor because its compound scaling strategy jointly balances network depth, width, and input resolution, providing a favorable trade-off between representational capacity and computational efficiency (Tan and Le, 2019). This compact architecture was considered suitable for generating deep embeddings from the segmented images in batches while using pretrained and frozen weights, without requiring the end-to-end training of a larger CNN architecture.

Prior to feature extraction, each segmented pig body image was converted to RGB format and resized to 224 × 224 pixels to match the spatial input dimensions used by EfficientNet-B0. Pixel values were converted into floating-point tensors scaled to the [0, 1] range and subsequently normalized channel-wise using the ImageNet mean values [0.485, 0.456, 0.406] and standard deviations [0.229, 0.224, 0.225]. EfficientNet-B0 was then used in feature extraction mode with ImageNet-pretrained weights. Its final classification layer was removed, the output of the global average pooling layer was retained, and all pretrained parameters were kept frozen. Through this forward pass, each segmented image was transformed into a 1,280-dimensional numerical feature vector.

The resulting 1,280-dimensional embedding constituted a distributed deep representation rather than a set of explicit manual morphometric measurements. It summarized convolutional activations that may encode information related to the overall silhouette, body contour, visible body proportions, posture, and texture, thereby abstracting complex visual patterns potentially associated with animal morphology and body weight (Li et al., 2021; Liu et al., 2023). The embeddings were generated in batches to ensure consistent processing across the dataset and were subsequently used as input variables for the supervised regression models.

#### 2.2.4 Analytical dataset construction and image–weight pairing

The feature vectors extracted from segmented images were integrated into a tabular structure suitable for supervised regression. Each feature vector was associated with its corresponding body weight, forming the analytical dataset used in this study.

The correspondence between images and weight values was established through a unique identifier assigned to each animal during data acquisition. This identifier was incorporated into file naming, ensuring consistent linkage between each embedding and its corresponding ground-truth weight.

As a result, a structured dataset was obtained in which each row represents a single instance, composed of a 1,280-dimensional feature vector and its associated target variable. This representation allowed the transformation of the weight estimation problem into a supervised regression task over tabular data (Li et al., 2021).

#### 2.2.5 Data splitting and validation strategy

Model performance was evaluated using a data partitioning strategy designed to ensure robustness and generalization. The dataset was divided into disjoint subsets for training, validation, and testing.

First, an independent test set corresponding to 15% of the total data was separated and reserved exclusively for final evaluation, preventing information leakage during training (Test set). The remaining data constituted the development set (TrainDev), used for model training and validation.

Within TrainDev, two additional subsets were defined: a tuning subset (Tune) and a cross-validation subset (CVPool). The Tune subset represented 15% of TrainDev and was used for hyperparameter optimization, while CVPool comprised the remaining data.

On CVPool, a stratified cross-validation scheme with 15 runs was implemented, allowing performance evaluation across multiple training and validation configurations and providing more stable estimates.

Stratification was performed using five body-weight strata derived from the observed distribution of the target variable through quantile-based binning. The resulting strata corresponded to the following observed weight ranges: 15–20 kg, 23 kg, 24 kg, 25–27 kg, and 29–42 kg. These strata were used exclusively to preserve the body-weight distribution across the data partitions, while body weight remained a continuous variable during model training and evaluation.

#### 2.2.6 Regression models

Body weight estimation was performed using supervised regression models applied to the tabular representations derived from deep features. Three representative approaches were evaluated: Support Vector Regression (SVR), XGBoost, and CatBoost, selected for their ability to model non-linear relationships in high-dimensional datasets (Chen et al., 2023; Valles-Coral et al., 2024).

SVR was used as a kernel-based approach capable of capturing complex relationships. Hyperparameter tuning resulted in the selection of a radial basis function (RBF) kernel, with a regularization parameter C=10.0 , an epsilon-insensitive loss parameter ∈=0.5 , and a scaling parameter γ="scale" , enabling appropriate control of model complexity.

XGBoost was implemented as a tree-based ensemble method using boosting techniques, modeling non-linear interactions through sequential error correction. The optimized configuration included 700 estimators, a learning rate of 0.03, a maximum depth of 5, and a minimum child weight of 2, along with subsampling strategies (subsample = 0.9; colsample_bytree = 0.9) and L1/L2 regularization to enhance generalization.

CatBoost was also evaluated as a boosting-based approach, known for improved training stability and strong performance in tabular regression tasks. The optimized configuration included 1,000 iterations, a learning rate of 0.03, a depth of 6, and an L2 regularization coefficient ( l2_leaf_reg=5.0 ), optimizing the RMSE loss function.

#### 2.2.7 Model performance evaluation

Model performance was assessed using standard regression metrics: Mean Absolute Error (MAE), Root Mean Squared Error (RMSE), and the coefficient of determination (R^2^). These metrics allow us to quantify the accuracy of the predictions from different perspectives, considering both the average magnitude of the error and the variability explained by the model (Terven et al., 2025).

Mean Absolute Error (MAE)

$$\mathrm{MAE}=\frac{\sum_{i=1}^{n}|y_{i}-\hat{y_{i}}|}{n}$$

Root Mean Squared Error (RMSE)

$$\text{RMSE}=\sqrt{\frac{1}{n}\sum_{i=1}^{n}{\left(y_{i}-\hat{y_{i}}\right)}^{2}}$$

Coefficient of determination (R^2^)

$$R^{2}=1-\frac{\sum_{i=1}^{n}{\left(y_{i}-\hat{y_{i}}\right)}^{2}}{\sum_{i=1}^{n}{\left(y_{i}-\bar{y}\right)}^{2}}$$

Among these, RMSE was considered the primary metric due to its sensitivity to large errors, which is particularly relevant in body weight estimation.

For statistical comparison between models, the Friedman non-parametric test for related samples was used, considering the RMSE values obtained by EfficientNet-B0 + SVR, EfficientNet-B0 + XGBoost, and EfficientNet-B0 + CatBoost across the same 15 cross-validation runs (Friedman, 1937). This approach allowed for the evaluation of overall differences in model performance.

Subsequently, a post-hoc analysis was performed using the Wilcoxon signed-rank test for the following pairwise comparisons: SVR versus XGBoost, SVR versus CatBoost, and XGBoost versus CatBoost (Wilcoxon, 1945). In each comparison, the RMSE values were paired according to the corresponding cross-validation run. Holm’s correction was applied to the resulting *p*-values to control the error associated with multiple comparisons (Holm, 1979). Additionally, Cohen’s dz. was calculated from the paired RMSE differences to quantify the magnitude of the observed differences (Lakens, 2013).

#### 2.2.8 Final evaluation on the test set

To assess the generalization capability of the models, a final evaluation was conducted using the independent test set (Test), which had been previously separated from the full dataset. This subset was not used during any stage of training, validation, or hyperparameter tuning, ensuring an unbiased evaluation of model performance, in line with recent studies on animal weight estimation using machine learning that rely on test sets to validate generalization ability (Chen et al., 2023).

The three evaluated models (SVR, XGBoost, and CatBoost) were trained using the complete development set (TrainDev) with the previously defined configurations and subsequently applied to the test set to obtain final predictions.

Model performance on the test set was primarily assessed using the RMSE metric, in order to maintain consistency with the evaluation conducted during cross-validation and to enable direct comparison between both scenarios, given that this metric is widely used in regression problems due to its sensitivity to large errors (Terven et al., 2025).

Additionally, a comparative analysis was performed between the RMSE distribution obtained during cross-validation and the value observed on the test set, with the aim of evaluating model stability and consistency when exposed to unseen data.

## 3 Results and discussion

### 3.1 Performance of the hybrid model based on segmentation and feature extraction

#### 3.1.1 Geometric normalization via reference marker detection

The application of the geometric normalization process enabled the standardization of image orientation and scale through the automatic detection of the circular reference marker, as illustrated in Figure 5. This procedure successfully adjusted the marker diameter to a uniform value in pixels, reducing variations associated with capture distance and framing.

**Figure 5:**
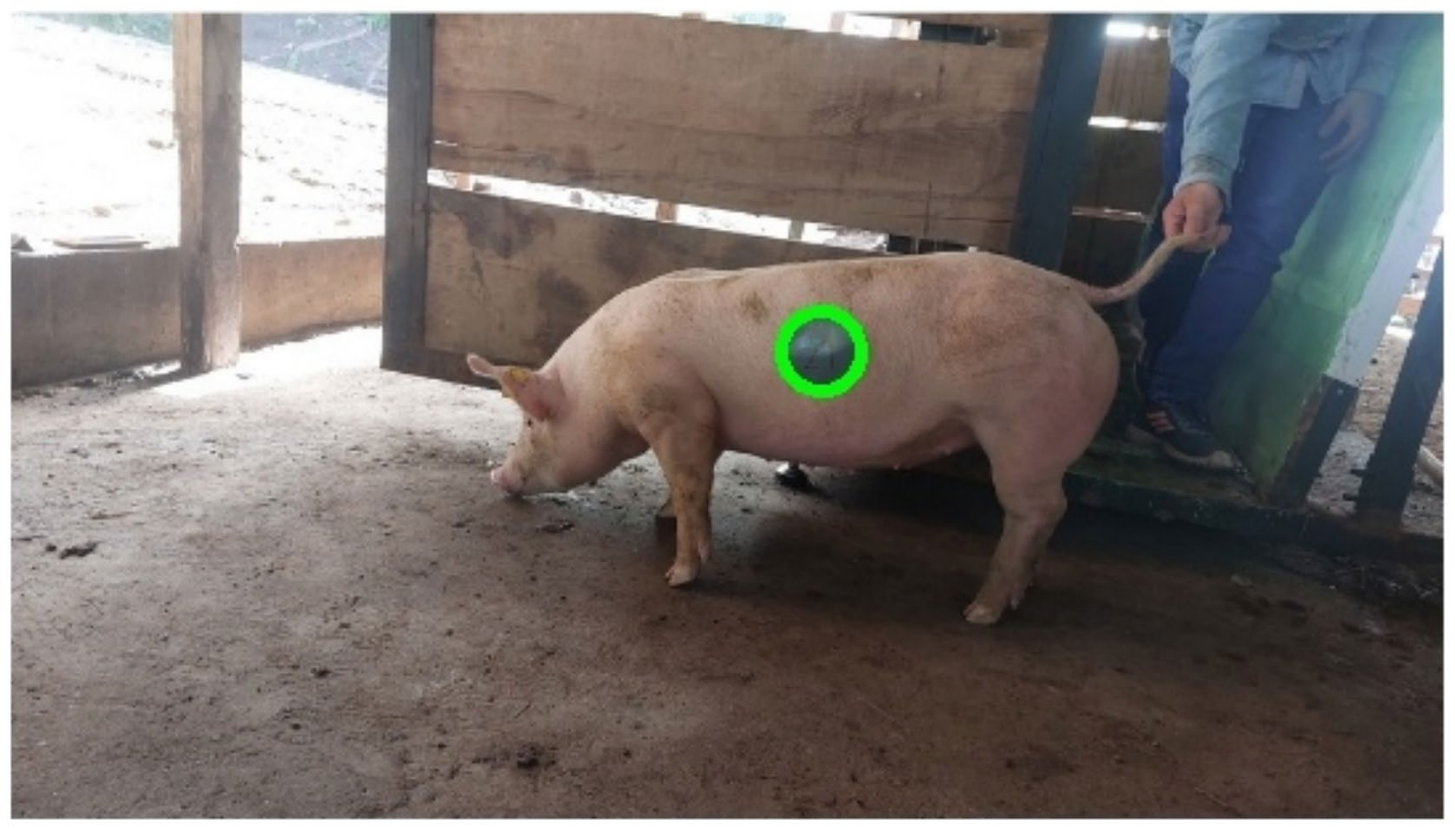
Reference marker detection and geometric normalization of the image.

As a result, a dataset with greater morphological consistency across samples was obtained, facilitating structural comparison between individuals and reducing visual variability not related to body weight. This standardization represents a key factor for improving the performance of computer vision models applied in real-world environments (Qin et al., 2022).

#### 3.1.2 Pig body segmentation

Quantitative evaluation on the validation subset of 46 images showed that the YOLOv8n-seg instance segmentation model achieved an mAP@0.5 of 97.5%, a precision of 98.7%, a recall of 93.5%, and an F1-score of 96.0%. These values correspond specifically to validation performance and were used to verify the adequacy of the segmentation model as an intermediate preprocessing component of the proposed pipeline. Following this validation stage, the exported segmentation checkpoint was applied to the 3,800 images of the study dataset to generate the pig-body masks used in the subsequent processing stages.

Figure 6 presents representative segmentation outputs selected from the study dataset after application of the trained model. These examples illustrate the model’s performance under the actual study conditions, including variations in lighting, background composition, and animal posture. The predicted masks delineated the visible pig body and reduced the influence of surrounding environmental elements before deep feature extraction. These quantitative results support the model’s ability to isolate the pig body under the evaluated farm conditions. In related livestock weight-estimation studies, Guvenoglu (2023) used semantic segmentation to extract cattle body-area information for live-weight prediction, whereas Peng et al. (2024) used YOLOv8-based yak detection and body-parameter extraction for real-time live-weight estimation.

**Figure 6:**
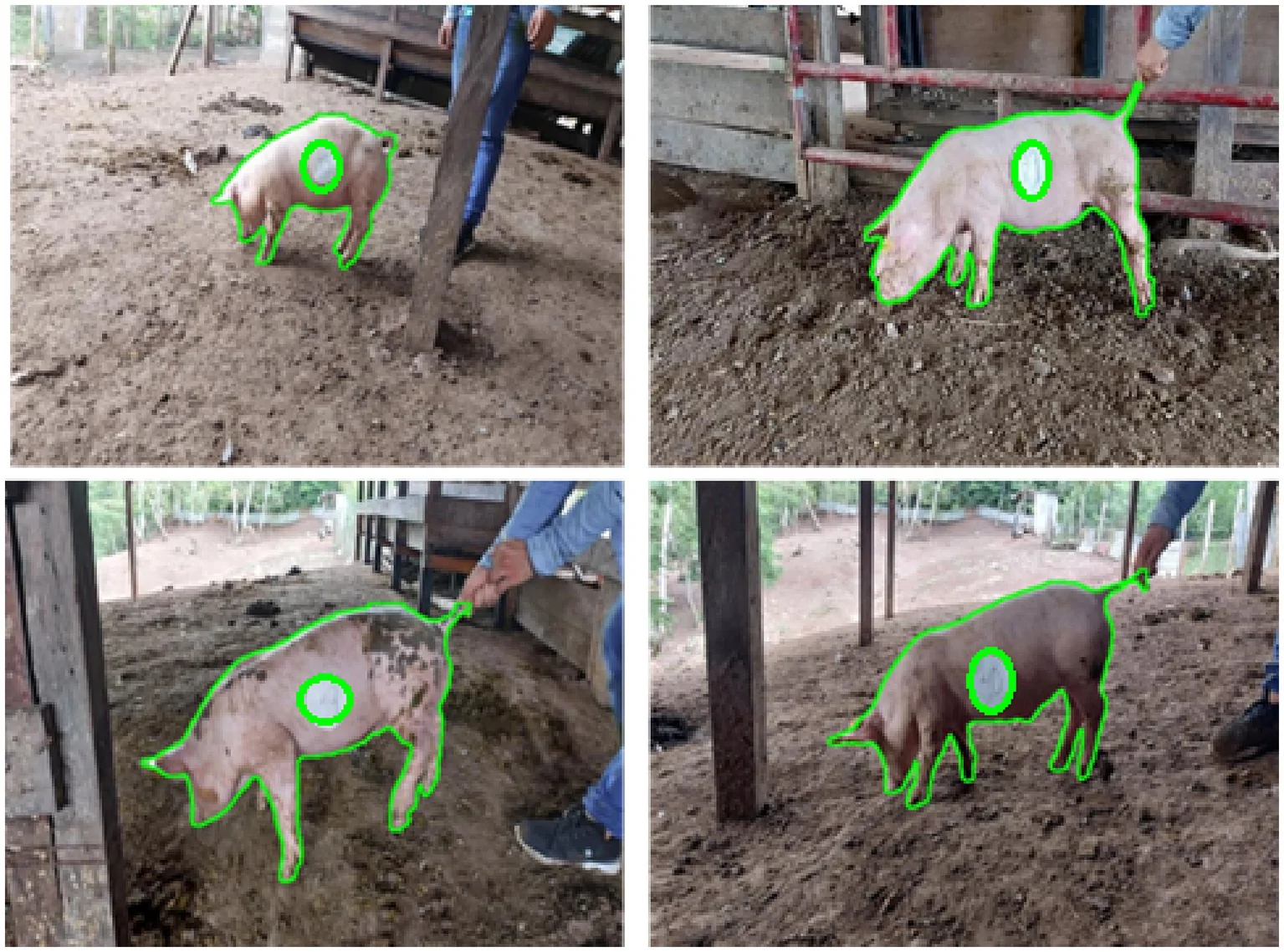
Results of pig body segmentation using YOLOv8n-seg.

### 3.2 Comparative analysis of regression models for weight estimation

#### 3.2.1 Individual performance of hybrid models

To analyze model performance across different validation partitions, Table 1 presents the values obtained for R^2^, MAE, and RMSE across the 15 cross-validation runs. These results allow for a detailed assessment of performance variability and enable a comprehensive comparison between models.

**Table 1:** Performance of the models in the 15 cross-validation iterations (R^2^, MAE and RMSE).

Group	EfficientNetB0 + SVR			EfficientNetB0 + XGBoost			EfficientNetB0 + CatBoost		
	R^2^	MAE	RMSE	R^2^	MAE	RMSE	R^2^	MAE	RMSE
1	0.8134	1.8344	3.0599	0.8180	1.7105	3.0214	0.8033	2.0448	3.1412
2	0.8364	1.6560	2.5037	0.6365	2.6442	3.7321	0.7390	2.1294	3.1622
3	0.9026	1.7424	2.5616	0.8929	1.6591	2.6868	0.8805	2.0901	2.8377
4	0.8056	2.0698	2.7661	0.6945	2.4844	3.4672	0.7200	2.3311	3.3194
5	0.8479	1.9209	2.7944	0.8168	1.8724	3.0669	0.8073	2.1184	3.1456
6	0.7626	2.3118	3.6127	0.7289	2.3844	3.8611	0.7375	2.5480	3.7993
7	0.8714	1.5419	2.2643	0.7562	1.9487	3.1179	0.7549	2.1360	3.1260
8	0.8904	1.3946	2.3310	0.8433	2.0157	2.7866	0.8700	1.8865	2.5382
9	0.9300	1.3715	1.9404	0.8265	1.9688	3.0552	0.8693	1.8477	2.6515
10	0.8491	1.9940	2.6673	0.7488	2.4773	3.4415	0.7941	2.3016	3.1154
11	0.9524	1.2343	1.5984	0.9219	1.4310	2.0478	0.8839	1.7741	2.4964
12	0.8616	1.9119	2.6408	0.7086	2.5709	3.8310	0.8170	2.1602	3.0357
13	0.7710	1.8243	3.0114	0.6596	2.4848	3.6708	0.7113	2.1023	3.3810
14	0.8563	1.7633	2.5734	0.7955	2.1272	3.0693	0.7995	1.9967	3.0396
15	0.7303	2.6996	3.9185	0.6565	2.9444	4.4225	0.6885	3.0529	4.2113

Based on the results presented in Table 1, the EfficientNetB0 + SVR model generally exhibits lower error values and higher coefficients of determination compared to the other approaches. On average, this model achieved an MAE of 1.8144 ± 0.3795, an RMSE of 2.6829 ± 0.5824, and an R^2^ of 0.8454 ± 0.0617, indicating a strong and reliable performance in body weight estimation.

In comparison, the EfficientNetB0 + CatBoost model demonstrated intermediate performance, with an average RMSE of 3.1334 ± 0.4473 and an R^2^ of 0.7917 ± 0.0649, maintaining relatively controlled variability across validation runs.

Meanwhile, the EfficientNetB0 + XGBoost model exhibited higher average error values (RMSE = 3.2852 ± 0.5801) and greater dispersion in its results, suggesting lower stability across different validation partitions.

#### 3.2.2 Statistical analysis of model performance

To comprehensively compare the performance of the evaluated models, RMSE was used as the primary metric due to its sensitivity to large errors, which is particularly relevant in body weight estimation. Based on the values obtained across the different validation partitions, a comparative statistical analysis was conducted to determine the existence of significant differences between models.

Table 2 presents the descriptive statistics calculated from the same 15 RMSE values obtained by each model across the cross-validation runs reported individually in Table 1. Overall, the SVR-based model achieved the lowest mean RMSE (M = 2.68), followed by CatBoost (M = 3.13) and XGBoost (M = 3.29). In addition, the SVR model exhibited a narrower confidence interval, suggesting greater consistency in its performance across validation runs.

**Table 2:** Descriptive statistics of RMSE by model.

Model	N	Mean	Std_Dev	Std_Error	95% confidence interval for the mean		Minimum	Maximum
					Lower limit	Upper limit		
EfficientNetB0 + SVR	15	2.682924	0.582371	0.150368	2.360417	3.005430	1.598380	3.918545
EfficientNetB0 + CatBoost	15	3.133372	0.447272	0.115485	2.885680	3.381063	2.496368	4.211313
EfficientNetB0 + XGBoost	15	3.285203	0.580118	0.149786	2.963945	3.606462	2.047835	4.422463

To evaluate the presence of statistically significant differences among the models, the non-parametric Friedman test for related samples was applied to the RMSE values obtained across the cross-validation runs. The results (Table 3) revealed significant differences between models (χ^2^ = 20.13, *p* < 0.001), with a large effect size (Kendall’s W = 0.671), indicating substantial agreement in the performance ranking.

**Table 3:** Friedman test for RMSE comparison among models.

Test	X^2^	gl	*p*	Kendall’sW
Friedman (paired)	20.133333	2	0.000042	0.671111

Following this result, a *post hoc* analysis was conducted using the Wilcoxon signed-rank test with Holm correction (Table 4). Pairwise comparisons revealed statistically significant differences between SVR and CatBoost (p_adj = 0.000183), as well as between SVR and XGBoost (p_adj = 0.000244). In contrast, no significant difference was found between CatBoost and XGBoost (p_adj = 0.094604).

**Table 4:** Post hoc comparisons using the Wilcoxon test with Holm correction.

Model A	Model B	Wilcoxon_w	p_raw	P_holm	Decision
EfficientNetB0 + CatBoost	EfficientNetB0 + SVR	0.0	0.000061	0.000183	Significant difference
EfficientNetB0 + SVR	EfficientNetB0 + XGBoost	1.0	0.000122	0.000244	Significant difference
EfficientNetB0 + CatBoost	EfficientNetB0 + XGBoost	30.0	0.094604	0.094604	Not significant

Effect size analysis (Table 5) further supports these findings, showing large effects for the comparisons between SVR and CatBoost (dz = 1.85) and between SVR and XGBoost (dz = −1.58), indicating substantial differences in performance. In contrast, the comparison between CatBoost and XGBoost showed a small-to-moderate effect size (dz = −0.49), suggesting a less pronounced difference between these models.

**Table 5:** Effect sizes (Cohen’s dz) for pairwise model comparisons.

Model A	Model B	Difference (A-B)	sd_diff	Cohen’s dz
EfficientNetB0 + CatBoost	EfficientNetB0 + SVR	0.450448	0.243667	1.848622
EfficientNetB0 + SVR	EfficientNetB0 + XGBoost	−0.602280	0.382132	−1.576106
EfficientNetB0 + CatBoost	EfficientNetB0 + XGBoost	−0.151832	0.309632	−0.490362

#### 3.2.3 Test set evaluation and stability analysis

Figure 7 presents the comparison between the RMSE distribution obtained during cross-validation and the corresponding RMSE value obtained on the independent test set for each evaluated model.

**Figure 7:**
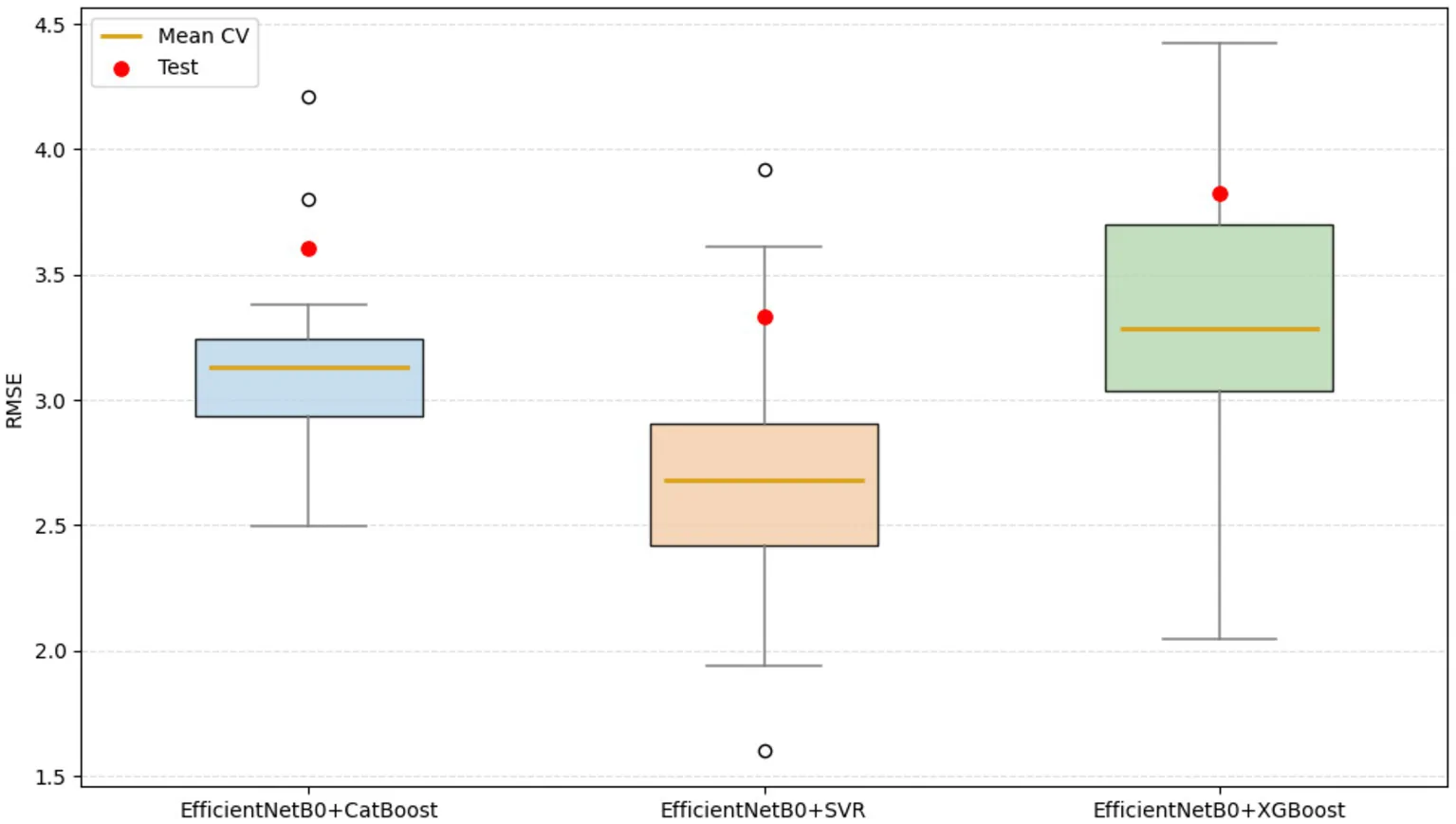
Comparison of RMSE between cross-validation and test set by model.

For the EfficientNetB0 + SVR model, the RMSE value obtained on the test set is close to the mean of the cross-validation distribution, indicating consistent behavior and strong generalization capability. Additionally, the relatively low dispersion of the values suggests stable performance across different data partitions.

For the EfficientNetB0 + CatBoost model, the RMSE value on the test set is slightly higher than the cross-validation mean, although it remains within the variability range of the distribution. This suggests that, while the model maintains overall coherent behavior, it experiences a slight decrease in performance when applied to unseen data.

In contrast, the EfficientNetB0 + XGBoost model exhibits greater dispersion in RMSE values during cross-validation, reflecting lower stability. In this case, the test RMSE also falls within the observed range but tends toward higher values, indicating a tendency to produce larger prediction errors compared to the other models.

Overall, the relationship between cross-validation and test set results indicates that all models maintain consistent behavior when applied to unseen data, with no clear evidence of overfitting. However, differences in stability and error magnitude are observed, with the SVR-based model demonstrating the highest consistency across both evaluation scenarios.

## 4 Discussion

The results obtained in this study demonstrate that the proposed hybrid approach enables accurate estimation of pig body weight under real farm conditions. In particular, the RMSE-based analysis and cross-validation results showed that the SVR model outperforms the boosting-based approaches, achieving lower average error and statistically significant differences. This behavior suggests that, within the generated deep feature space, kernel-based methods are more effective at capturing the non-linear relationships between animal morphology and body weight.

This finding is particularly relevant, as it partially contrasts with recent benchmark studies in which gradient-boosted decision-tree models, including XGBoost and CatBoost, have demonstrated strong and competitive performance on tabular datasets (Gorishniy et al., 2021; Grinsztajn et al., 2022; McElfresh et al., 2023). These studies also show that no single model family is universally superior and that relative performance depends on the characteristics of the dataset. In the present study, the lower RMSE values obtained by SVR suggest that the EfficientNet-B0 feature representation was more suitable for the kernel-based regression approach than for the evaluated boosting models.

On the other hand, the CatBoost model exhibited relatively stable behavior, reflected in lower RMSE dispersion compared to XGBoost. Nevertheless, the statistical analysis showed that this greater stability did not translate into lower overall prediction error than that obtained with the SVR model.

Regarding the earlier stages of the pipeline, the incorporation of geometric normalization through a reference marker effectively reduced spatial variability across images, improving the consistency of morphological representations. Such strategies have been identified as critical in computer vision systems applied in real-world environments, where variability in acquisition conditions can significantly impact model performance (Qin et al., 2022).

Similarly, YOLOv8-based segmentation proved effective in isolating the region of interest even under conditions of variable lighting and heterogeneous backgrounds, in line with recent reports highlighting the robustness of such architectures in livestock applications (Peng et al., 2024; Wang et al., 2024). Accurate delineation of the animal’s silhouette contributed to improving the quality of the generated embeddings, reinforcing the importance of segmentation as a critical stage within the pipeline.

From a comparative perspective, the results are consistent with studies employing computer vision for animal weight estimation, which report that the combination of segmentation and deep learning can reduce prediction error (Chen et al., 2023; Guvenoglu, 2023). Unlike approaches that rely on 3D sensors or specialized devices, the present study achieved competitive predictive performance using RGB images captured with a mobile device, providing an accessible alternative for small- and medium-scale production systems. This practical advantage is relevant because cost and implementation complexity remain important barriers to the adoption of precision livestock technologies (Busch, 2023; Siegford, 2024). However, the observed tendency toward underestimation in higher weight ranges indicates a limitation of two-dimensional images in representing the full volumetric characteristics of the animal, as also reported in previous studies (Liu et al., 2023; Paudel et al., 2023). Future research could therefore explore the integration of additional information sources to improve performance in these specific scenarios without compromising the practical applicability of the system.

## 5 Conclusion

This study demonstrates that a hybrid approach based on instance segmentation, deep feature extraction, and supervised regression enables accurate and non-invasive estimation of pig body weight under real farm conditions. The results show strong predictive performance, with the SVR model emerging as the most accurate and consistent approach according to RMSE-based statistical analysis.

The use of RGB images captured with mobile devices, combined with an efficient processing pipeline, positions the proposed method as an accessible and practical solution for implementation in small- and medium-scale production systems. In addition, its non-invasive nature contributes to improved animal welfare and enhanced data quality.

Overall, these findings highlight the potential of hybrid models in precision livestock farming applications, providing a foundation for the development of more robust systems through the integration of multimodal approaches and validation in diverse production environments.

## Data Availability

The dataset used in this study is not publicly available at this stage because it is being prepared for formal publication as a separate data paper. However, it may be made available upon reasonable request to the corresponding author for academic and verification purposes.
